# Nuclear factor I-C disrupts cellular homeostasis between autophagy and apoptosis via miR-200b-Ambra1 in neural tube defects

**DOI:** 10.1038/s41419-021-04473-2

**Published:** 2021-12-20

**Authors:** Wanqi Huang, Tianchu Huang, Yusi Liu, Jialin Fu, Xiaowei Wei, Dan Liu, Wei Ma, Hui Gu, Zhengwei Yuan

**Affiliations:** Key Laboratory of Health Ministry for Congenital Malformation, Shengjing Hospital, China Medical University, Shenyang, China

**Keywords:** Neurulation, Disease genetics

## Abstract

Impaired autophagy and excessive apoptosis disrupt cellular homeostasis and contribute to neural tube defects (NTDs), which are a group of fatal and disabling birth defects caused by the failure of neural tube closure during early embryonic development. However, the regulatory mechanisms underlying NTDs and outcomes remain elusive. Here, we report the role of the transcription factor nuclear factor I-C (NFIC) in maintaining cellular homeostasis in NTDs. We demonstrated that abnormally elevated levels of NFIC in a mouse model of NTDs can interact with the miR-200b promoter, leading to the activation of the transcription of miR-200b, which plays a critical role in NTD formation, as reported in our previous study. Furthermore, miR-200b represses autophagy and triggers apoptosis by directly targeting the autophagy-related gene *Ambra1* (Autophagy/Beclin1 regulator 1). Notably, miR-200b inhibitors mitigate the unexpected effects of NFIC on autophagy and apoptosis. Collectively, these results indicate that the NFIC-miR-200b-Ambra1 axis, which integrates transcription- and epigenome-regulated miRNAs and an autophagy regulator, disrupts cellular homeostasis during the closure of the neural tube, and may provide new insight into NTD pathogenesis.

## Introduction

Neural tube defects (NTDs) are a group of severe congenital malformations of the central nervous system, typified by spina bifida, anencephaly, and encephalocele, which are caused by incomplete closure of the neural tube during embryonic development [1, 2]. It is estimated that ~300,000 infants are diagnosed with NTDs each year [3]. Individuals with NTDs are mostly stillborn, die shortly after birth, or suffer from varying degrees of disabilities [4, 5]. Comprising a series of complex polygenic diseases, NTDs are caused by the synergistic interaction of environmental and genetic factors [6]. However, current research into the molecular mechanisms underlying the pathogenesis of NTDs is just the tip of the iceberg.

Dysregulation of cellular homeostasis occurs via the disruption of autophagy and the activation of apoptosis, which can lead to abnormal closure of the neural tube during the development of the fetal central nervous system [7]. Autophagy is an evolutionarily advantageous process in which autophagosomes are fused with lysosomes to degrade unwanted cytoplasmic components [8–10]. The ability of autophagy to remove damaged cellular components is restricted in NTDs [11, 12]. Furthermore, in a recent study, we determined that mitophagy in the spinal cord is inhibited in all-trans retinoic acid (ATRA)-induced spina bifida and that resveratrol treatment can reduce spina bifida formation by ameliorating mitophagy impairment; these findings indicate that autophagy is causally implicated in NTDs [13]. In addition, excessive apoptosis of neuroepithelial cells leads to an insufficient number of cells within the neural folds, which is another major factor in the formation of NTDs [14, 15]. Similarly, in a previous study, we found that excessive apoptosis in neuroepithelial cells aggravates the formation of ATRA-induced NTDs [16]. Notably, autophagy helps relieve cellular endoplasmic reticulum stress and sustain cellular homeostasis and cell viability, thereby inhibiting apoptosis [11, 17]. Growing evidence has indicated that impaired autophagy and enhanced apoptosis contribute to intracellular imbalance and ultimately lead to neurulation failure [8, 9]. Therefore, identifying the potential risk factors of unbalanced cellular homeostasis during the closure of the neural tube is crucial for developing novel preventive therapies for NTDs.

In the neural system of embryos with NTDs, numerous dysregulated miRNAs that are strongly involved in NTD formation have been identified [18, 19]. For example, in our previous study, miRNAs detected in maternal serum were considered potential biomarkers for the noninvasive prenatal diagnosis of NTDs [20, 21]. In addition, our research group reported the miR-200b-induced silencing of CITED2 mediated excessive apoptosis of neural stem cells caused by high glucose, leading to NTDs [22]. As a member of the miR-200 family, miR-200b participates in a variety of molecular biological functions in the neural system during embryo development. Recently, increasing evidence has illustrated the negative regulation of autophagy by miR-200b via reduction of ATG12 and ATG5 expression in human lung adenocarcinoma and breast cancer cells, respectively [23, 24]. Nevertheless, it remains unclear whether miR-200b regulates autophagy during neural tube development. The autophagy-related gene *Ambra1* (Autophagy/Beclin regulator 1, encoding the activating molecule in Beclin1-regulated autophagy) is also thought to be closely related to NTDs; its functional deficiency in mouse embryos leads to severe NTDs [25, 26]. Rare mutations in *Ambra1* may also contribute to human NTDs [27]. According to bioinformatics findings, Ambra1 is a potential target of miR-200b [22]; therefore, we speculate that miR-200b not only plays a role in regulating apoptosis but also in managing autophagy through Ambra1 in NTDs.

Although the aberrant regulation of miR-200b has been implicated in the formation of NTDs [22], definitive evidence explaining this dysregulation is lacking, and the molecules mediating miR-200b have not previously been characterized during embryonic neurulation. It has been documented that the expression of miR-200b can be regulated by several transcriptional factors via binding to the promoter of miR-200b. For instance, GRHL2 regulates the expression of miR-200 family genes to maintain epithelial plasticity and stemness in oral cancer cells by directly activating miR-200 promoters [28]. Therefore, this study explores the transcriptional factor regulating miR-200b during embryonic neural development. According to existing databases (TransmiR v2.0, JASPAR, hTFtarget) and further investigation, we determined that the nuclear factor I-C (NFIC) is the relevant transcriptional factor of miR-200b. NFI has been shown to regulate transcription in multiple organ-system development processes by controlling cell proliferation and differentiation, especially in the central nervous system [29–32]. However, no previous study has investigated the role of NFIC in NTDs. In this study, we hypothesized that NFIC impairs autophagy and triggers apoptosis by modulating miR-200b-Ambra1 during NTD formation.

Herein, we explore the molecular mechanisms underlying the role of NFIC and miR-200b in impeding autophagy and exacerbating apoptosis, which ultimately leading to NTDs; moreover, we investigated the effects of the downregulation of Ambra1 via the transcriptional activation of miR-200b by NFIC. Our results revealed that the NFIC-miR-200b pathway silences Ambra1, thereby disrupting cellular homeostasis via impaired autophagy and excessive apoptosis, and consequently, resulting in the failure of neural tube closure. The proposed NFIC-miR-200b-Ambra1 axis provides a basis for potential therapeutic interventions for NTDs.

## Materials and methods

### Animal models

Female C57BL/6J mice aged 8–10-weeks old were obtained from the Animal Center of Shengjing Hospital of China Medical University (Shenyang, Liaoning, China). An ATRA-induced mouse model of NTDs was prepared as described in our previous study [16, 33]. Male and female mice were paired overnight, and pregnancy was established by the presence of the vaginal plug the next morning; the noon of that day was designated as embryonic day 0.5 (E0.5). Pregnant mice were divided into two groups: one group was treated with ATRA by gavage (70 mg/kg body weight; Sigma-Aldrich, St. Louis, MO, USA) and the other group (the control group) was only treated with olive oil on an embryonic day 8.5 (E8.5). On E9.5, pregnant mice were euthanized, and embryos were dissected out of uteri using a dissecting microscope (SZH-ILLB, Olympus, Kyoto, Japan). All procedures for animal use adhered to the National Institute of Health Guide for the Care and the animal ethics committee of Shengjing Hospital of China Medical University (Approval No. 2016PS113K).

### Cell culture and transfection

C17.2 mouse neural stem cells purchased from Beina Chuanglian Biology Research Institute were maintained in a minimum essential medium (MEM, Gibco, MA, USA) supplemented with 10% fetal bovine serum (FBS, Gibco, MA, USA), 1% MEM non-essential amino acids (MEM NEAA, Gibco, MA, USA) and 100 U/ml penicillin, and 100 µg/ml streptomycin in a humidified incubator at 37 °C with 5% CO_2_. C17.2 cells were seeded into six-well plates with 1 × 10^5^ cells/well and cultured for 24 h before transfection. Transfections were performed according to manufacturers’ protocols using JetPRIME (Polyplus Transfection, Illkirch, France).

### Reagents and plasmid construction

The miR-200b mimic/inhibitor and control mimic/inhibitor oligos were purchased from GenePharma, (Shanghai, China), and RIBOBIP (Guangzhou, China). SiRNAs against NFIC were synthesized by RIBOBIP (Guangzhou, China). Biotin-labeled miR-200b was custom designed and purchased from Dharmacon (CO, USA). The coding sequences for the mRNAs of NFIC were cloned into the pcDNA3.3 vector. The 2-kb sequence of the miR-200b promoter immediately upstream of the transcription start sites (TSS) was cloned into the pGL4-basic vector (Liaoning Bai Hao Biological Technology Co. Ltd, China). The 3ʹ-UTR of Ambra1, as well as the wild-type (WT) and mutated (Mut) form of the predicted miR-200b binding site, were amplified and subcloned into the pmirGLO Dual-Luciferase miRNA Target Expression Vector to generate the 3ʹ-UTR-Luc, WT-Luc, and Mut-Luc reporters, respectively (Liaoning Bai Hao Biological Technology Co. Ltd, China). Primers for miR-200b, U6, NFIC, Ambra1, and β-actin were produced by Genescript (Nanjing, China); all primer sequences are provided in Supplementary Table 1.

### Dual-luciferase reporter assay

The pGL4 containing different promoter sequences of miR-200b was cotransfected into C17.2 cells with or without the NFIC plasmid. The indicated Ambra1 luciferase reporter was cotransfected into C17.2 with the miR-200b mimic or the control mimic. Cells were harvested after 48 h, and cell lysates were collected for tests of firefly and Renilla luciferase activities measured using the dual-luciferase reporter assay system (Promega, WI, USA) according to the manufacturer’s protocol and normalized by the Renilla luciferase activities.

### Chromatin immunoprecipitation (ChIP) assay

Chromatin immunoprecipitation (ChIP) assays were performed using the SimpleChIP Enzymatic Chromatin IP Kit (Cell Signaling Technology, #9005, MA, USA) according to the manufacturer’s recommended protocol. In brief, C17.2 cells were crosslinked with formaldehyde and lysed with cell lysis buffer followed by sonication to an average size of 150–900 bp. Thereafter, chromatin extracts were immunoprecipitated using the anti-NFIC antibody (Cell Signaling Technology, #11911, MA, USA); negative control was performed using normal rabbit IgG (Cell Signaling Technology, #2729, MA, USA) overnight. The purified DNA was subjected to quantitative real-time polymerase chain reaction (RT-qPCR) analysis to amplify the binding sites of the miR-200b promoter region.

### Biotin-labeled miR-200b pulldown assay

The biotin-labeled negative control or biotin-labeled miR-200b was transfected into C17.2 cells after 48 h, and the cell lysate was collected. Next, streptavidin-coupled Dynabeads (Invitrogen, CA, USA) were added into the cell lysate and incubated at 4 °C on a rotator overnight. After washing the beads thoroughly, the purified RNA was obtained and used for qRT-PCR analyses. The input RNA was then extracted to serve as the control.

### Immunoblotting

E9.5 mouse embryos or C17.2 cells were lysed in RIPA buffer (Beyotime, Shanghai, China), and the total protein levels were measured using a BCA kit (Takara, Ohtsu, Japan). Immunoblotting was performed as previously described [16]. After blocking the membranes (Millipore, MA, USA), they were incubated with the primary antibodies against NFIC (1:1000, Invitrogen, CA, PA5-90204), Ambra1 (1:1000, Cell Signaling Technology, #24907, MA, USA), LC3 (1:1000, Cell Signaling Technology, #4108, MA, USA), Cleaved-caspase 3 (1:1000, Cell Signaling Technology, #9664, MA, USA), Bax (1:1000, Cell Signaling Technology, #2772, MA, USA), Bcl-2 (1:1000, Sigma-Aldrich, B9804, MO, USA), and β-actin (1:3000, Proteintech, 66009-1-lg, Wuhan, China) overnight at 4 °C. The next day, the membranes were incubated with secondary HRP-conjugated antibodies (1:5000, Proteintech, Wuhan, China) at room temperature for 2 h. A chemiluminescent substrate (Millipore, MA, USA) was used to visualize the signals. Protein bands were analyzed using Image J and normalized to β-actin levels.

### miRNA and RNA extraction and RT-qPCR

The total mRNA from embryos and C17.2 cells was extracted with TRIZOL (Takara, Ohtsu, Japan), and the miRNA was extracted with the miRNeasy Mini Kit (Qiagen, CA, USA) according to the manufacturer’s instructions. Complementary DNA (cDNA) was synthesized using a PrimeScript™ RT reagent kit (Takara, Ohtsu, Japan) and a miRNA First Strand cDNA Synthesis (Takara, Ohtsu, Japan) with RNA as the template. mRNA and miRNA expression levels were measured with the SYBR Premix Ex Taq kit (Takara, Ohtsu, Japan) on a 7500 Real-time PCR system (StepOnePlus, ABI Company, Oyster Bay, NY, USA). The relative expression of genes was calculated using the 2^−△△Ct^ method. The primers for candidate genes, β-actin, and U6 are shown in Supplementary Material.

### Immunofluorescence staining

Cells were seeded in a 12-well plate with cell slides and transfected for 48 h in different treatment conditions. Cells were washed with PBS and fixed with 4% paraformaldehyde, and then incubated in 0.5% Triton. After blocking with 1% donkey serum, the cells were incubated with the primary antibody: rabbit anti-LC3 (1:100, Cell Signaling Technology, #4108, MA, USA) overnight at 4 °C, and subsequently with the secondary antibody conjugated to Alexa Fluor 488 (1:100, Cell Signaling Technology, #8878, MA, USA). Nuclei were counterstained with DAPI (4’,6-diamidino-2-phenylindole). Images were observed under laser scanning confocal microscopy (Zeiss, Oberkochen, Germany).

### Apoptosis and caspase 3 activity determination

Cells were seeded in a 24-well plate and transfected for 48 h. Caspase 3 expression was analyzed using the GreenNuc kit (Beyotime, Shanghai, China) according to the manufacturer’s instructions. Images were obtained using a fluorescence microscope (Nikon EclipseTi, Nikon ECLIPSE 80i, Kyoto, Japan).

### TUNEL assay

TUNEL assay was performed using an in-situ cell death assay kit, TMR red (Roche, Mannheim, Germany), according to the manufacturer’s protocol. Briefly, cells were seeded onto an 8-well glass slide (Thermo Fisher Scientific, MA, USA) and transfected. Thereafter, cells were fixed, permeabilized, and incubated with the TUNEL reagent. DAPI staining was performed for recognition of nuclei and categorization of cells. Apoptotic cells were visualized and imaged using a fluorescence microscope (Nikon ECLIPSE 80i, Kyoto, Japan).

### Statistical analysis

All experiments were performed independently with at least three biological replicates. Statistical analyses and graph generation were performed using GraphPad Prism 8.0 software, and data are presented as the mean ± SEM. Statistical comparison between two groups was conducted using the Student’s *t* test, whereas that among three or more groups was performed using one-way analysis of variance (ANOVA). *P* < 0.05 was considered statistically significant.

## Results

### miR-200b and NFIC expression were increased in the NTD mouse model

**Fig. 1:**
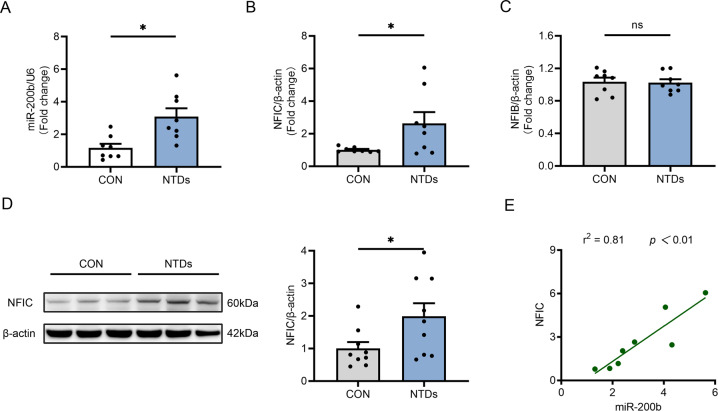
Expression of miR-200b and NFIC in a mouse model of ATRA-induced NTDs. **A** Relative expression of miR-200b in normal and ATRA-induced NTD embryos at E9.5 (*n* = 8). mRNA levels of NFIC (**B**) and NFIB (**C**) in E9.5 embryos from control and NTD groups (*n* = 8). **D** Protein abundance of NFIC in E9.5 mouse embryos (*n* = 9). **E** RNA expression correlation analysis for miR-200b and NFIC in NTD mouse embryos, as shown in the panel. The bar graph shows quantitative data from all experiments, which were independently performed in triplicate. *P < 0.05 vs. control group, “ns” represents no significance, compared to the control group.

Our previous study demonstrated that miR-200b levels were increased by maternal diabetes or high glucose in vitro, which is critical for endoplasmic reticulum homeostasis and NTD formation in developing embryos [22]. Consistently, miR-200b levels were strongly increased in the mouse model of ATRA-induced NTDs at E9.5 compared with those in control embryos (Fig. 1A). However, the mechanism of miR-200b upregulation in NTDs remains unknown. Previous studies have shown that the promoter of miR-200b can be recognized by transcription factors, which enhance the transcription of miR-200b, which inspires us to speculate that the enrichment of miR-200b in NTDs is due to the regulation of transcription factors. We preliminarily targeted NFIB and NFIC as potential transcription factors of miR-200b, identified using website databases (JASPAR, hTFtarget, TransmiR V2.0). These transcription factors belong to the NFI family and have site-specific DNA-binding functions in the modulation of gene expression [34]. Furthermore, RT-qPCR verification revealed that the mRNA expression of NFIC (Fig. 1B) but not NFIB (Fig. 1C) was increased in NTD embryos; the protein abundance of NFIC was also upregulated in NTD embryos (Fig. 1D). Therefore, we chose NFIC as the transcription factor of miR-200b for further analysis. Moreover, miR-200b showed a strong positive correlation with the RNA expression of NFIC in NTD embryos (*r*^2^ = 0.81) (Fig. 1E), indicating activated regulation between NFIC and miR-200b.

### miR-200b expression was transcriptionally activated by NFIC

**Fig. 2:**
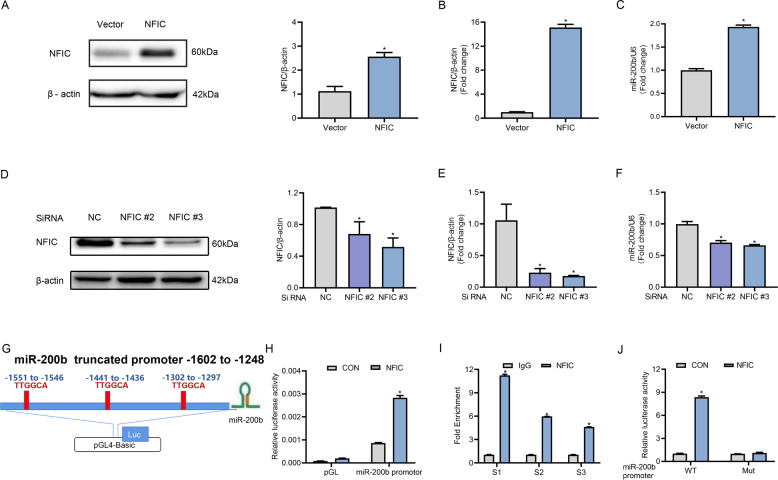
NFIC positively regulates transcription of miR-200b. Changes in protein expression (**A**) and mRNA expression (**B**) of NFIC after transfection of the NFIC plasmid or the control vector in C17.2 neural stem cells. **C** RT-qPCR analysis of miR-200b levels in cells transfected with the NFIC plasmid or the control vector. Levels of NFIC protein (**D**) and mRNA (**E**) after transfecting the NFIC SiRNA or the control SiRNA into cells. **F** miR-200b levels after silencing NFIC with SiRNA in C17.2 cells. **G** A diagram showing the construct of the miR-200b promoter-luciferase reporter encompassing three binding motifs of TTGGCA. **H** The miR-200b promoter construct was cotransfected with the NFIC plasmid or the control vector into cells. Luciferase activity was assayed 48 h after transfection. **I** ChIP analysis of NFIC binding to the three conserved motifs in the promoter of miR-200b (S1, S2, and S3). **J** Luciferase activity was measured after cotransfection of the WT or Mut type of the miR-200b promoter with or without the NFIC plasmid in C17.2 cells. The results represent three repeated experiments. *P < 0.05 vs. control group.

Next, we investigated the modulation of NFIC on miR-200b. Upon overexpressing NFIC in the C17.2 mouse neural stem cell line, significantly higher expression of miR-200b and NFIC was observed (Fig. 2A–C). Next, we utilized three SiRNAs against NFIC to silence NFIC (supplementary Fig. 1). Among these, SiNFIC 2# and SiNFIC 3# were selected for further experiments as they decreased the protein expression of NFIC more effectively than SiNFIC 1#. Similarly, miR-200b levels were reduced accordingly when endogenous NFIC expression was silenced by transfection with NFIC SiRNA (Fig. 2D–F). Collectively, these results indicate that miR-200b is a downstream target of the transcription factor NFIC and was upregulated by NFIC.

It remains unclear whether NFIC-enhanced miR-200b expression occurs via a transcriptional mechanism. Therefore, we cloned a 2-kb fragment of the promoter region of miR-200b and inserted it into the pGL4-reporter luciferase construct to determine the promoter activity of miR-200b (Fig. 2G). The results showed that overexpression of NFIC resulted in remarkably increased luciferase activity in cells cotransfected with the miR-200b promoter (Fig. 2H). There are three conserved motifs of TTGGCA in the promoter of miR-200b (S1, S2, and S3), which are predicted NFIC binding sites according to the JASPAR online tool (Fig. 2G). To further determine whether NFIC could bind directly to the miR-200b promoter and to characterize the specific bind site, endogenous ChIP assays were performed in C17.2 cells. As shown in Fig. 2I, compared with the negative control (IgG), NFIC combined with the binding sites in the miR-200b promoter, especially in S1. Moreover, in order to confirm the reliability of our results, we subcloned the WT and Mut type of the miR-200b promoter encompassing -1602 bp to -1248 bp relative to TSS, which contains all three binding sites. The luciferase reporter driven by the WT miR-200b promoter generated dramatically higher levels of luciferase activity in cells transduced with NFIC. In contrast, mutation of the miR-200b promoter did not affect luciferase activity when NFIC was cotransfected in cells (Fig. 2J). These findings imply that the transcription factor NFIC interacted with the miR-200b promoter directly to promote the transcription of miR-200b.

### miR-200b interacted with Ambra1 mRNA and suppressed Ambra1 expression

**Fig. 3:**
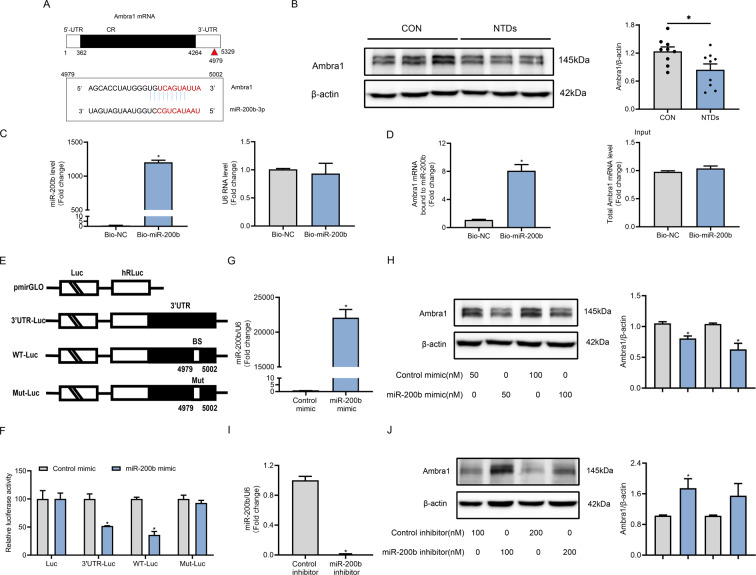
miR-200b binds to 3ʹ-UTR of Ambra1 mRNA and inhibits Ambra1 translation. **A** Schematic representation of Ambra1 mRNA, depicting a potential miR-200b binding site (BS) located in Ambra1 3′-UTR. **B** Western blot of Ambra1 in E9.5 mouse embryos and quantification of Ambra1 protein in the control and NTD groups (*n* = 9). **C** Abundance of miR-200b and U6 examined by RT-qPCR after 48 h of biotin-miR-200b transfection. **D** Ambra1 mRNA levels detected in the lysates precipitated by biotin-miR-200b and the total input. **E** Schematic of different Ambra1 luciferase reporter plasmids. Luc Luciferase, BS miR-200b-binding site, WT wild-type, Mut mutation. **F** Relative luciferase activity levels of 3ʹ-UTR-Luc, WT-Luc, and Mut-Luc reporters after miR-200b transfection. Levels of miR-200b (**G**) and Ambra1 protein (**H**) in C17.2 cells transfected with the indicated dose of the miR-200b mimic or the control mimic for 48 h. Changes in miR-200b (**I**) and Ambra1 protein (**J**) expression after transfection of different doses of the miR-200b inhibitor or the control inhibitor. *P < 0.05 vs. control group.

miRNAs modulate the expression of target mRNAs by either inhibiting translation or promoting degradation [35]. Based on this, we attempted to identify the direct target of miR-200b that mediates neural tube development. According to TargetScan and miRbases, Ambra1 was predicted as a putative downstream target of miR-200b (Fig. 3A). Ambra1 is required for embryonic neurulation, and its deficiency impairs autophagy and aggravates apoptosis, resulting in NTDs [36]. In this study, we found the Ambra1 expression was suppressed in the mouse model of ATRA-induced NTDs (Fig. 3B). To verify *Ambra1* as a target gene of miR-200b, we performed an RNA pull-down assay with biotin-labeled miR-200b, which revealed that Ambra1 mRNA levels were remarkably increased after transfecting the biotin-labeled miR-200b (Fig. 3C, D). Moreover, we conducted luciferase reporter assays to determine the interaction between miR-200b and Ambra1 mRNA by transfecting the 3ʹ-UTR-Luc reporter, which contained the potential binding site of miR-200b (Fig. 3E). Next, to further verify the specific miR-200b binding sites in the Ambra1 3ʹ-UTR, we subcloned the WT-Luc and Mut-Luc of the putative binding site into the luciferase reporter vector (Fig. 3E). The results showed that miR-200b repressed the luciferase reporter intensity of the 3ʹ-UTR-Luc and WT-Luc reporters, but not that of the Mut-Luc reporter (Fig. 3F).

Next, the regulation of Ambra1 by miR-200b was further validated by transfecting the mimic or inhibitor of miR-200b into C17.2 cells. The protein levels of Ambra1 were inhibited by 50 nmol/L or 100 nmol/L of miR-200b mimic (Fig. 3G, H), whereas the mRNA levels were unaffected (Supplementary Fig. 2). In contrast, the miR-200b inhibitor upregulated the protein expression of Ambra1 (Fig. 3I, J) but not its mRNA expression (Supplementary Fig. 2). These data imply that miR-200b silenced Ambra1 protein expression by directly binding to Ambra1 mRNA and blocking its translation in NTDs.

### NFIC induced autophagy impairment via activation of the miR-200b-Ambra1 circuit

**Fig. 4:**
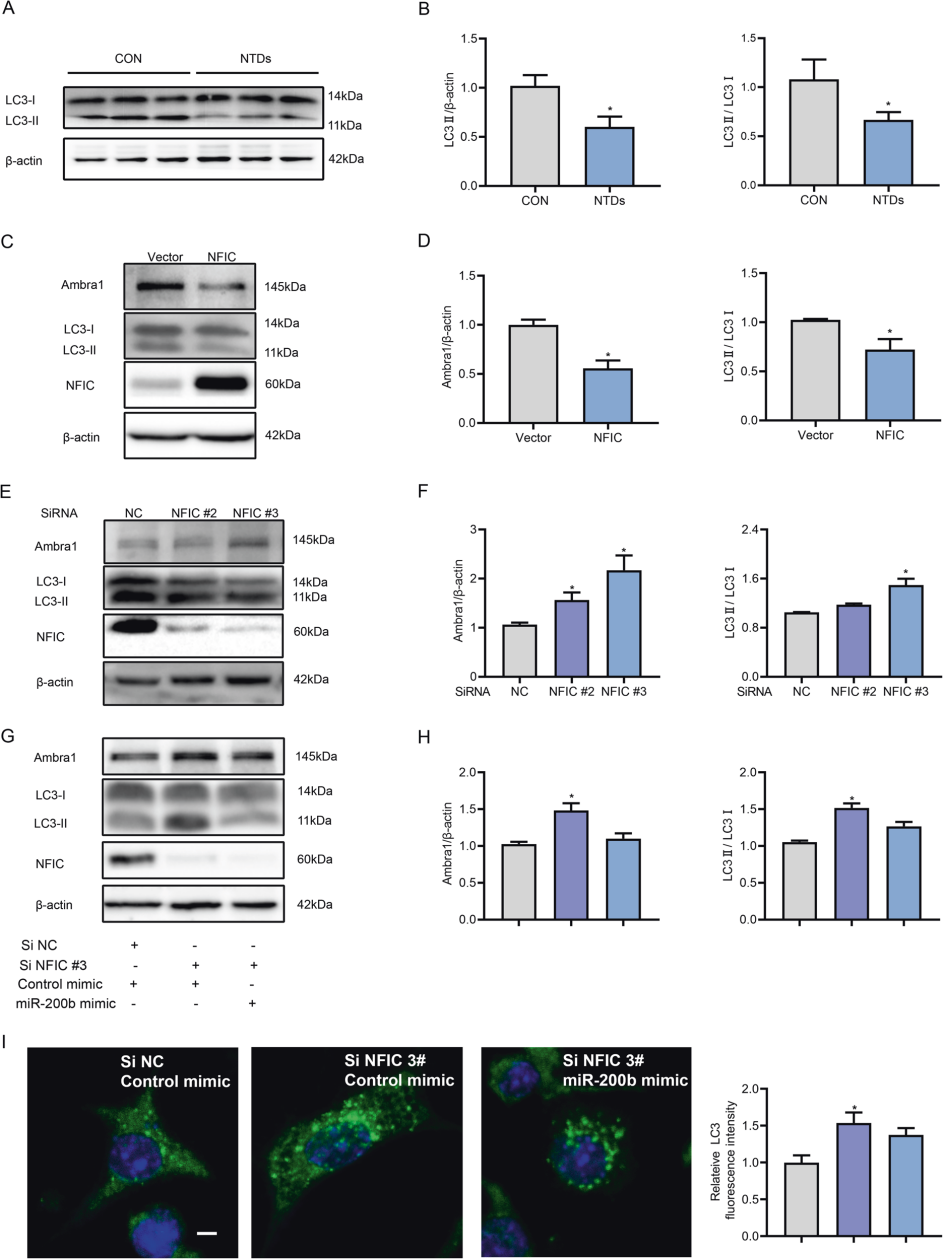
miR-200b rescues NFIC knockdown-induced autophagy. **A**, **B** Protein expression of LC3 in normal and ATRA-induced NTD embryos (*n* = 3). **C**, **D** Protein levels of Ambra1, LC3, and NFIC after C17.2 cells were transfected with the NFIC plasmid or the control vector. **E**, **F** Effect of NFIC SiRNA on the protein expression of Ambra1, LC3, and NFIC. **G**, **H** Ambra1, LC3, and NFIC protein expression in cells transfected with the NFIC SiRNA and the control mimic or the miR-200b mimic. **I** The fluorescence intensity of LC3 in cells transfected with the NFIC SiRNA and the control mimic or the miR-200b mimic, scale bar = 5 µm. Experiments were repeated three times. *P < 0.05 vs. control group.

Consistent with the findings of our previous study [13], a biochemical parameter for autophagy (the conversion of LC3-I to LC3-II) was repressed in the mouse model of ATRA-induced NTDs (Fig. 4A, B). We then explored the functional consequence of NFIC overexpression in NTDs on autophagy. Upon overexpressing NFIC in cultured C17.2 cells, Ambra1 and LC3-II expression was evidently weakened (Fig. 4C, D). In contrast, NFIC SiRNA promoted the upregulation of Ambra1 as well as LC3-II (Fig. 4E, F). The abundance of Ambra1 and conversion of LC3-I to LC3-II by NFIC SiRNA were abrogated by miR-200b (Fig. 4G, H). Consistent with the above results, NFIC SiRNA significantly increased LC3 puncta, which were abolished by miR-200b (Fig. 4I). Collectively, these results demonstrate that NFIC resulted in autophagy impairment by enhancing the miR-200b-Ambra1 circuit in neural stem cells.

### NFIC-triggered cell apoptosis was mediated by miR-200b

**Fig. 5:**
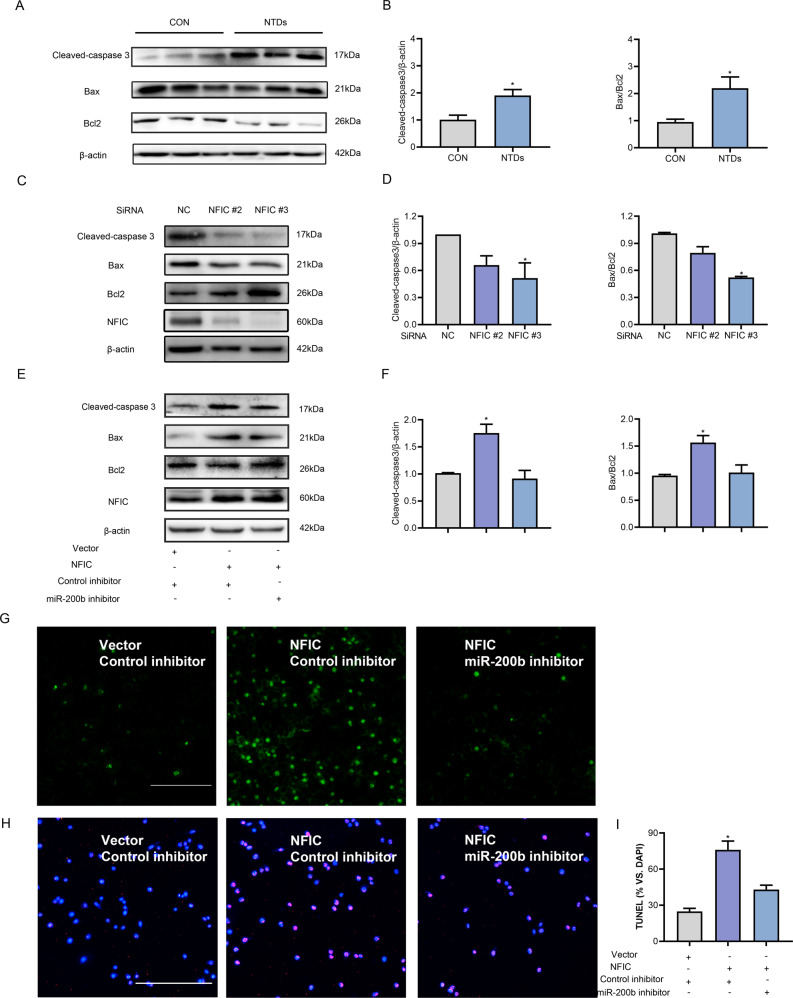
NFIC-miR-200b circuit triggers apoptosis in C17.2 cells. **A**, **B** Protein levels of apoptosis markers (Cleaved-caspase 3, Bax, and Bcl-2) in E9.5 NTD mouse embryos compared with the control group (*n* = 3). **C**, **D** Protein expression of Cleaved-caspase 3, Bax, Bcl-2, and NFIC estimated by western blot in cells transfected with the NFIC SiRNA or the control SiRNA. **E**, **F** Changes in Cleaved-caspase 3, Bax, Bcl-2, and NFIC proteins after transfection of the NFIC plasmid with or without the miR-200b inhibitor. Apoptotic cells are shown by representative confocal microscopic images of C17.2 cells cotransfected with the NFIC plasmid and the miR-200b inhibitor or control inhibitor, using the active caspase 3 assay, scale bar = 100 µm (**G**) and TUNEL assay (**H**, **I**), scale bar = 100 µm. Data from three replicate experiments are shown in the bar graphs. *P < 0.05 vs. control group.

Impaired autophagy leads to an increase in apoptosis [8], and excessive apoptosis is a key factor in the formation of NTDs [36]. In agreement with the findings of our previous study [16], the expression of pivotal apoptosis-related proteins, such as Cleaved-caspase 3, Bax, and Bcl-2, was altered in the mouse model of ATRA-induced NTDs (Fig. 5A, B). To determine the effect of NFIC on neural tube closure through apoptosis, the expression of apoptosis-related proteins, along with the number of apoptotic cells, was quantified. We observed that the silencing of NFIC inhibited the expression of the pro-apoptotic proteins Cleaved-caspase 3 and Bax but increased the expression of the anti-apoptotic protein Bcl-2 (Fig. 5C, D). Next, we verified whether NFIC-induced cell apoptosis was mediated via the miR-200b-Ambra1 circuit. The overexpression of NFIC increased the abundance of pro-apoptotic proteins, whereas the miR-200b inhibitor reversed the NFIC-induced upregulation of pro-apoptotic proteins (Fig. 5E, F). Furthermore, NFIC-induced apoptosis was ameliorated by Ambra1 overexpression (Supplementary Fig. 3). Caspase 3 expression is an important indicator of cell apoptosis. Therefore, we estimated the number of apoptotic cells with the GreenNuc kit, which indicates the expression of active caspase 3 with green fluorescence. The cells with NFIC overexpression exhibited obvious fluorescence; however, the suppression of miR-200b could alleviate the NFIC-induced cell apoptosis (Fig. 5G). The TUNEL assay verified this rescue effect of the miR-200b inhibitor on apoptosis (Fig. 5H, I). Thus, our data suggest that NFIC promoted cell apoptosis by activating miR-200b-Ambra1, leading to embryonic NTD formation.

## Discussion

**Fig. 6:**
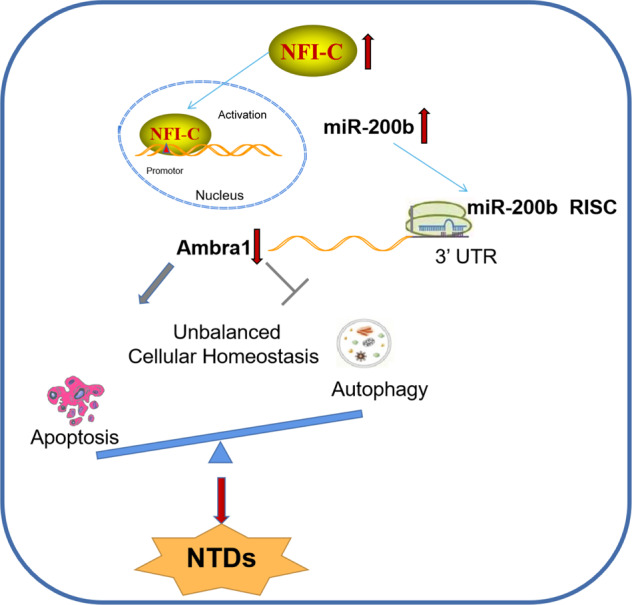
Role of NFIC-miR-200b-Ambra1 pathway in modulating autophagy and apoptosis in NTDs. A schematic diagram depicting the overexpression of NFIC-enhanced miR-200b transcriptional activity, followed by the suppressed translation of Ambra1, which is the downstream target of miR-200b, leading to impaired autophagy/excessive apoptosis and the subsequent formation of NTDs.

Despite recent progress in elucidating the etiology of NTDs, the precise mechanisms underlying NTDs are far from understood. This study is the first to identify that NFIC, a transcriptional factor, is abnormally elevated in NTDs and appears to play a key role in neural tube closure. Specifically, the overexpression of NFIC stimulated miR-200b, which reduced Ambra1 expression by binding to its mRNA and inhibiting translation, and in turn, led to impaired autophagy, excessive apoptosis, and subsequent NTD formation. Conversely, blocking of miR-200b-Ambra1 obviously rescued autophagy deficits and apoptosis during neural tube closure (Fig. 6). These results highlight the role of the NFIC-miR-200b-Ambra1 axis in managing autophagy and apoptosis in NTDs and provide a mechanistic basis for embryonic neural development.

It is well documented that NFIC is a key gene required for tooth root formation [37]. NFIC is also broadly involved in the development of mammalian neural tissue. Moreover, genome-wide association studies have revealed that genetic variants of NFIC are also associated with Alzheimer’s disease [38, 39]. Furthermore, recent studies have demonstrated that NFIC is related to the role of the transcription factor in regulating AD genesis and development [40, 41]. In addition, through RNA-seq gene expression profiling of the brain, NFIC has been shown to regulate differentially expressed genes in autism spectrum disorder [42]. However, the current study is the first to demonstrate the critical role of NFIC in impeding the closure of the neural tube. We revealed that the overexpression of NFIC in NTDs repressed autophagy and triggered apoptosis mediated by Ambra1. NFIC has previously been proven to stimulate apoptosis by activating p53 in the mouse mammary gland in the mid-pregnancy period [43]. However, no studies have elucidated the effect of NFIC in modulating autophagy. Our study clarified the vital role of NFIC in modulating autophagy and apoptosis for the formation of NTDs. In addition, NFIC knockout mice have been reported to exhibit unique tooth pathologies and significant dental defects [44], verifying that the restriction of odontoblast proliferation and differentiation due to NFIC deficiency could contribute to dental defects [45, 46]. Therefore, NFIC could stimulate cell proliferation and differentiation, with the excessive proliferation of neuroepithelial cells and premature differentiation of neural progenitor cells disrupting neural tube closure during the early stage of neurulation [47, 48]. Thus, future studies should explore the effect of NFIC on cell proliferation and differentiation during embryonic neurulation.

As NFIC is a transcription factor, the transcriptional regulation of coding RNAs by NFIC has been widely investigated [49]. The diverse range of the target substrates of NFIC confers its multiple biological functions. For instance, NFIC can promote osteoglycin expression by binding to its promoter region to repress bladder cancer by limiting cell proliferation and invasiveness [50]. Nevertheless, the transcriptional role of NFIC on miRNA is still uncertain. NFIC is known to recognize and bind to the TTGGCA motifs [51]. Notably, the miR-200b promoter harbored three TTGGCA regions. The luciferase reporter assay and ChIP assay in mouse neural stem cells further confirmed that NFIC could directly interact with the TTGGCA motifs in the miR-200b promoter and positively activate the transcription of miR-200b. Thus, our findings support the key transcriptional role of NFIC in non-coding RNA.

In addition, miR-200b has recently emerged as a key regulator of neurogenesis and gliogenesis in adult neural homeostasis in the central nervous system [52]. Our previous study revealed that miR-200b is overexpressed in diabetes-induced NTDs and elucidated the stimulative effect of miR-200b through CITED2 (CBP/p300-interacting transactivator with Glu/Asp-rich C-terminal domain, 2) on neural stem cell apoptosis [22]. Similarly, in the current study, miR-200b was augmented in ATRA-induced NTDs. Accumulating evidence has indicated the involvement of miR-200b in the modulation of autophagy: that is the upregulation of miR-200b inhibits cell autophagy, which leads to the repression of esophageal fibrosis or chemoresistance in human lung adenocarcinoma [23, 53]. However, no previous study has investigated the effect of miR-200b on autophagy in NTDs. Here, we discovered Ambra1 as a novel target of miR-200b, which binds to the 3ʹ-UTR of *Ambra1* at the site of 4979–5002 bp to block the translation of Ambra1. Ambra1 is a crucial positive regulator of autophagy initiation [54]; therefore, we proposed a novel mechanism whereby elevated miR-200b repressed autophagy by inhibiting Ambra1, which contributed to the formation of NTDs. Furthermore, although Ambra1 is specifically expressed in the neural plate of mouse embryos during the early stages of neurulation [25, 26], and Ambra1 deficiency occurs in NTDs [27, 55], to our knowledge, no previous study has explained the reason for the deficiency of Ambra1 in NTDs.

Cellular homeostasis mechanisms have significant roles in preventing cellular damage that can lead to impairment of cellular function, and ultimately, cell death. Autophagy and apoptosis are critical for maintaining cellular environmental homeostasis during embryo development; they can be regulated by common mediators and exhibit multiple routes for crosstalk [56]. Our study elucidated that the deficiency of Ambra1 induced by NFIC-activated miR-200b promotes impaired autophagy and excess apoptosis, subsequently leading to cellular homeostatic imbalance; this indicates that Ambra1 plays a role in modulating autophagy and apoptosis in NTDs. The cellular function of Ambra1 lies at the crossroads between autophagy and apoptosis, which decides cell death or survival. The functional loss of *Ambra1* leads to a significant increase in apoptosis [26, 57]. Moreover, Ambra1 deficiency increased susceptibility to different apoptotic stimuli [58, 59]. On the other hand, caspase and calpain pathways result in the degradation of Ambra1 and other autophagic proteins [58]. This indicates a positive feedback loop between autophagy and apoptosis, and implies a considerable role of Ambra1 in cellular homeostasis. In our study, apart from Ambra1, LC3-II (a marker of autophagosomes) also exhibited lower levels in NTD mouse embryos, whereas pro-apoptosis markers showed higher levels. Thus, Ambra1 deficiency, resulting in impaired autophagy, could aggravate apoptosis in the neuroepithelium, thereby disrupting the cellular homeostasis that is required for the closure of the neural tube.

In summary, this study provided evidence for the differential expression of NFIC, miR-200b, and Ambra1 in NTD embryos and elucidated a novel pathway comprising the NFIC-miR-200b-Ambra1 axis, which modulates NTD formation by inhibiting autophagy and triggering apoptosis. Our findings provide novel insights into the pathogenesis of NTDs and suggest potential therapeutic targets for NTDs. Nevertheless, to better guide future investigations, some limitations need to be noted. One of these is the lack of in vivo experiments involving NFIC in the mouse model of NTDs. Future work should explore the preventive effect of silencing NFIC on embryonic development in NTDs. Moreover, future research efforts should use the birth cohort established by our research group to further confirm the expression of key factors in neural tube closure in human NTD specimens, thereby providing a better theoretical basis for developing future therapeutic targets.

## Supplementary information

supplementary Table 1

supplementary figure legends

supplementary figure 1

supplementary figure 2

supplementary figure 3

Checklist

Authors contribution

## Data Availability

Data sharing is not applicable to this article as no new data were generated or analyzed in this study.
