# Supplementary material for: Nuclear factor I-C disrupts cellular homeostasis between autophagy and apoptosis via miR-200b-Ambra1 in neural tube defects
Source: Cell Death Dis. 2021 Dec 20;13(1):17. doi: 10.1038/s41419-021-04473-2 (PMC8688449; doi:10.1038/s41419-021-04473-2)
Supplement: Supplementary file 1 — supplementary Table 1 [file 41419_2021_4473_MOESM1_ESM.doc]

Supplementary Table 1 The sequences of primers used in qRT-PCR

| Genes | Primers | Sequence (5’-3’) |
| --- | --- | --- |
| β-actin | Forward | GGAGATTACTGCCCTGGCTCCTA |
|  | Reverse | GACTCATCGTACTCCTGCTTGCTG |
|  |  |  |
| U6 | Forward | CTCGCTTCGGCAGCACA |
|  | Reverse | AACGCTTCACGAATTTGCGT |
|  |  |  |
| miR-200b-3p | Forward | GAATACTGCCTGGTAATGATGACAA |
|  | Reverse | GCTGTCAACGATACGCTACGTAAC |
|  |  |  |
| NFIC | Forward | CCGGCATGAGAAGGACTCTAC |
|  | Reverse | TTCTTCACCGGGGATGAGATG |
|  |  |  |
| Ambra1 | Forward | CCAGAGAAGAATGCTGTACGAAT |
|  | Reverse | TCCATCGAGTCTTATCCTCCAC |
|  |  |  |
| RNA pull down Ambra1 | Forward | GAAATAGCCTGCTTGCCTGAC |
|  | Reverse | CACATTCTGACCTGGAATCTCC |
|  |  |  |
| ChIP S1 | Forward | CCTTTAAATTTTGCTGGAGGACC |
|  | Reverse | AACATGGGAGGCTAGGGGACT |
|  |  |  |
| ChIP S2 | Forward | CCTAAGAAGTGCTCACCCTG |
|  | Reverse | GAATTTCAGTTATCCTTGTGGC |
|  |  |  |
| ChIP S3 | Forward | GTCCACTTAGTTCTCAAATAG |
|  | Reverse | TCTCAAACACCCGTCAGATA |

S：Site
