# Supplementary material for: Nuclear factor I-C disrupts cellular homeostasis between autophagy and apoptosis via miR-200b-Ambra1 in neural tube defects
Source: Cell Death Dis. 2021 Dec 20;13(1):17. doi: 10.1038/s41419-021-04473-2 (PMC8688449; doi:10.1038/s41419-021-04473-2)
Supplement: Supplementary file 2 — supplementary figure legends [file 41419_2021_4473_MOESM2_ESM.doc]

Figure 1

Levels of NFIC protein after transfecting the SiRNAs NFIC 1#, 2#, 3# or the control SiRNA into cells.

Figure 2.

**(A, B)** mRNA expression of Ambra1 after transfection of the miR-200b mimic/inhibitor or the control mimic/inhibitor in C17.2 neural stem cells.

Figure 3.

Levels of Cleaved-caspase 3 protein after cotransfecting with the NFIC and Ambra1 /vector plasmid into cells.
