# Supplementary figures and images for: Nuclear factor I-C disrupts cellular homeostasis between autophagy and apoptosis via miR-200b-Ambra1 in neural tube defects
Source: Cell Death Dis. 2021 Dec 20;13(1):17. doi: 10.1038/s41419-021-04473-2 (PMC8688449; doi:10.1038/s41419-021-04473-2)

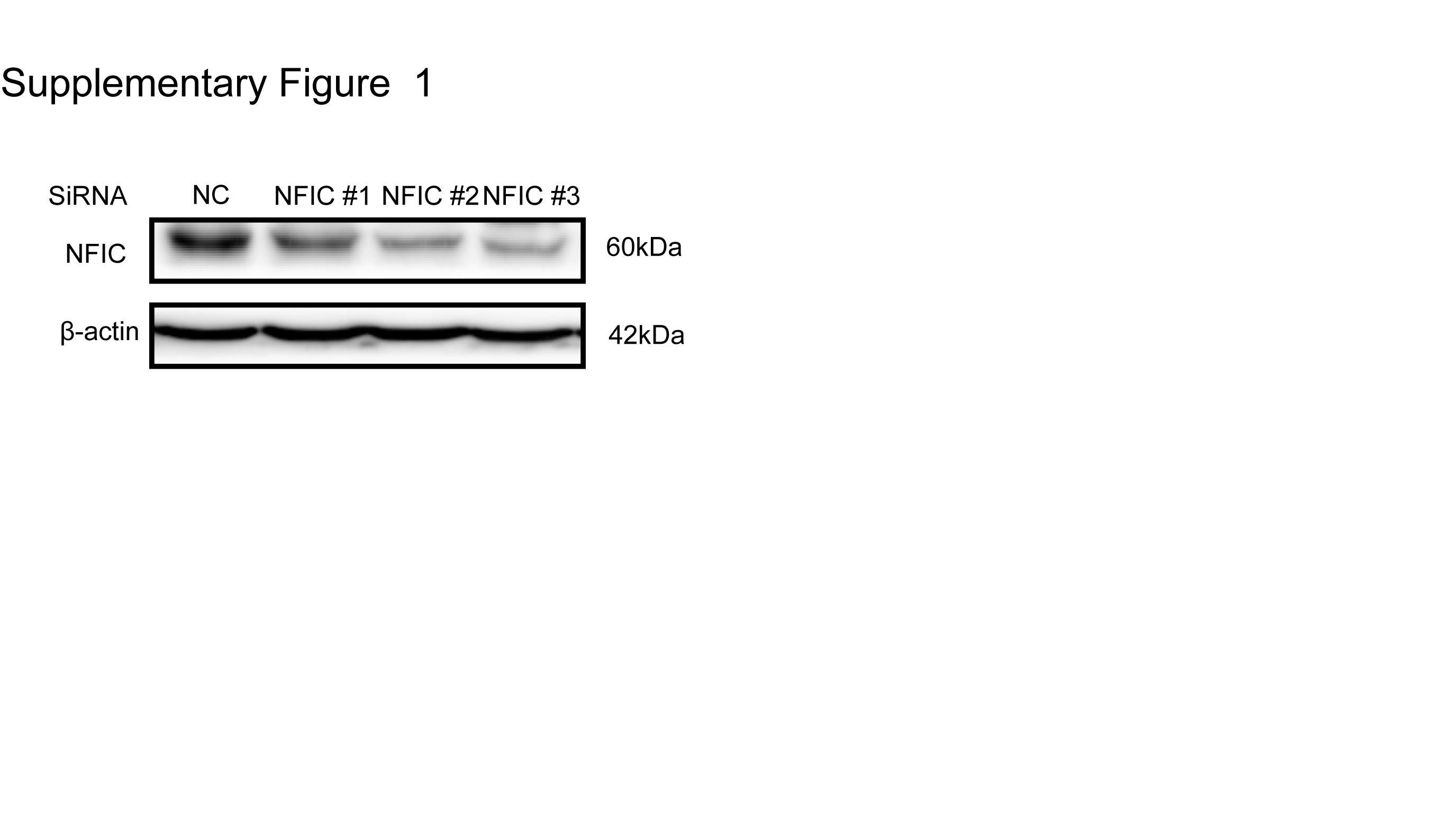

Supplement: Supplementary file 3 — supplementary figure 1 [file 41419_2021_4473_MOESM3_ESM.tif]

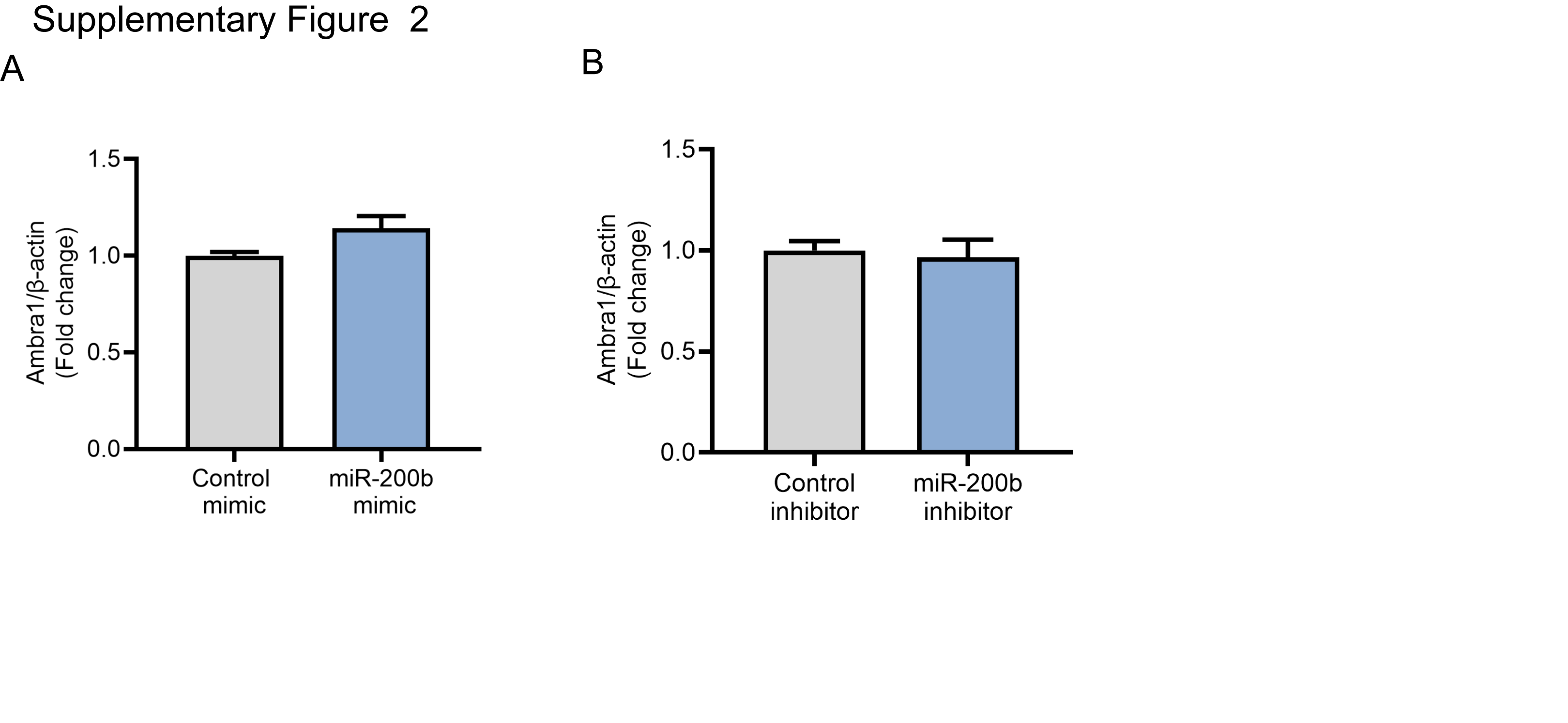

Supplement: Supplementary file 4 — supplementary figure 2 [file 41419_2021_4473_MOESM4_ESM.tif]

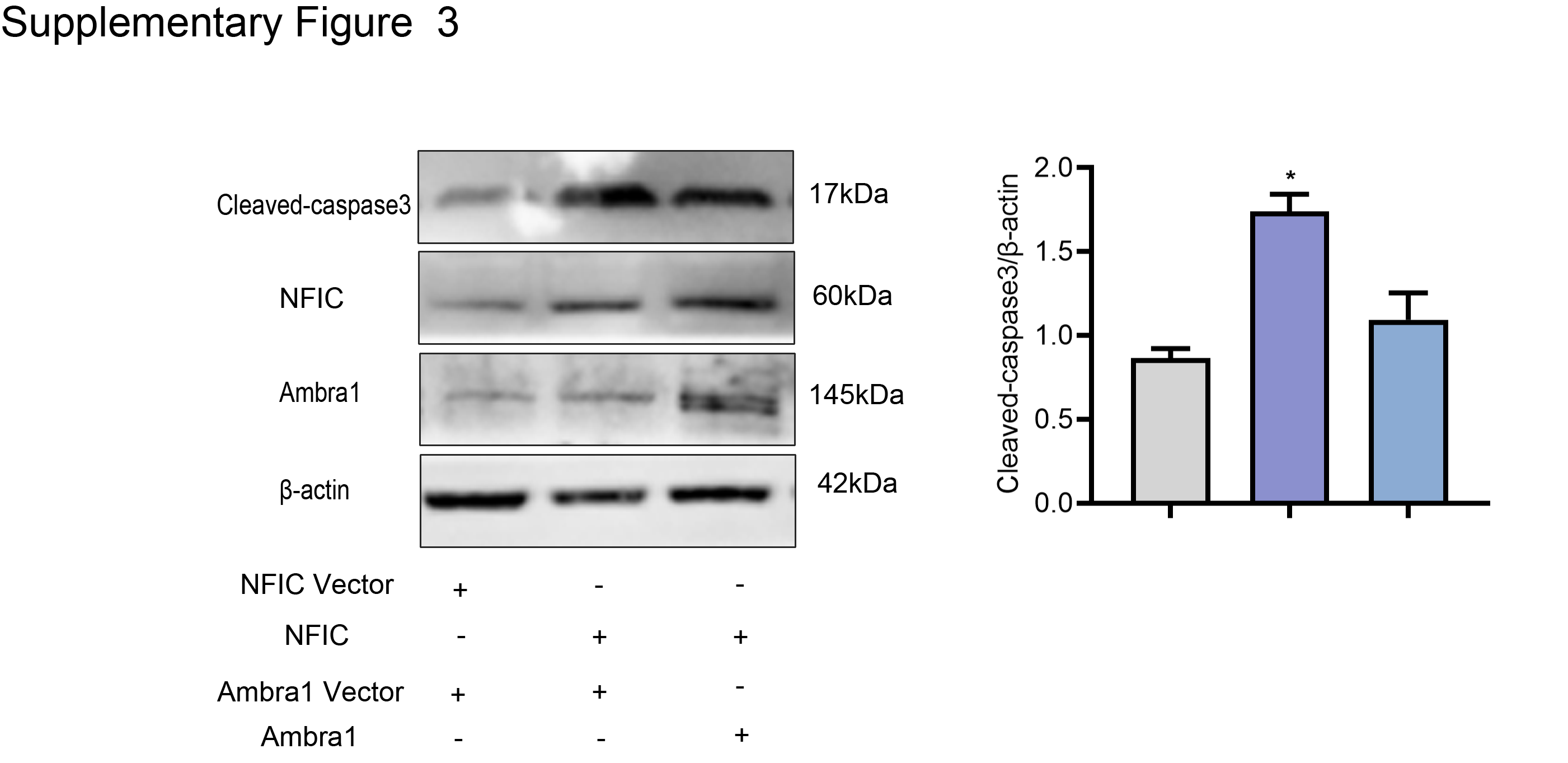

Supplement: Supplementary file 5 — supplementary figure 3 [file 41419_2021_4473_MOESM5_ESM.tif]
