# Supplementary material for: Nuclear factor I-C disrupts cellular homeostasis between autophagy and apoptosis via miR-200b-Ambra1 in neural tube defects
Source: Cell Death Dis. 2021 Dec 20;13(1):17. doi: 10.1038/s41419-021-04473-2 (PMC8688449; doi:10.1038/s41419-021-04473-2)
Supplement: Supplementary file 7 — Authors contribution [file 41419_2021_4473_MOESM7_ESM.pdf]

DECLARATION OF CONTRIBUTIONS TO ARTICLE **ADMC**

Manuscript Number: CDDIS-21-3761R

Journal Name: *Cell Death & Disease* (the 'Journal')

Proposed Title of the Contribution:

Nuclear factor I-C disrupts cellular homeostasis between autophagy and apoptosis via miR-200b-Ambra1 in neural tube defects (the 'Contribution)

Author(s):

Wanqi Huang, Hui Gu, Tianchu Huang, Yusi Liu, Jialin Fu, Xiaowei Wei, Dan Liu, Wei Ma, and Zhengwei Yuan (the 'Authors')

For all *CDDis* articles, each person named as an author in the published version must be able to show he or she has contributed substantially to the article.

Authorship credit should be based on 1) substantial contributions to conception and design, acquisition of data, or analysis and interpretation of data; 2) drafting the article or revising it critically for important intellectual content; and 3) final approval of the version to be published. Authors should meet conditions 1, 2 and 3.

Any person who cannot be shown to have made a substantial contribution to the article cannot be listed as an author in the final version. The name of any person who is deemed to have made a minor contribution can, however, appear in the Acknowledgments section of the article.

Please complete the table below to indicate the contributions of all named authors to the manuscript.

Author Full Name:	Specification of Contribution to the Manuscript:
Wanqi Huang	Designed the study, performed the experiments, analyzed the data, and drafted the article
Hui Gu	Designed the study, performed the experiments, analyzed the data, drafted the article and final approval of the version to be published.
Tianchu Huang	Performed the experiments, collected and analyzed the data
Yusi Liu	Performed the experiments, collected and analyzed the data
Jialin Fu	Performed the experiments, collected and analyzed the data
Xiaowei Wei	Collected and analyzed the data and revised the article
Dan Liu	Collected and analyzed the data and revised the article
Wei Ma	Collected and analyzed the data and revised the article
Zhengwei Yuan	Helped to design the study and revised the article

Please complete the table below to indicate the contributions of all named authors to the figures.

Figure 1:
Wanqi Huang, Hui Gu, Tianchu Huang

Figure 2:
Wanqi Huang, Hui Gu,Yusi Liu

Figure 3:
Wanqi Huang, Hui Gu, Xiaowei Wei, Dan Liu

Figure 4:
Wanqi Huang, Hui Gu, Tianchu Huang, Jialin Fu, Wei Ma

Figure 5:
Wanqi Huang, Hui Gu, Tianchu Huang, Yusi Liu

Figure 6:
Wanqi Huang, Hui Gu, Zhengwei Yuan

Signed for and on behalf of the Author(s):	Print Name:	Date:
Hui Gu	Hui Gu	11/25/2021
